# Transcriptomic Analysis of the Effects of Chemokine Receptor CXCR3 Deficiency on Immune Responses in the Mouse Brain during *Toxoplasma gondii* Infection

**DOI:** 10.3390/microorganisms9112340

**Published:** 2021-11-12

**Authors:** Kousuke Umeda, Youta Goto, Kenichi Watanabe, Nanako Ushio, Ragab M. Fereig, Fumiaki Ihara, Sachi Tanaka, Yutaka Suzuki, Yoshifumi Nishikawa

**Affiliations:** 1 National Research Center for Protozoan Diseases, Obihiro University of Agriculture and Veterinary Medicine, Obihiro 080-8555, Hokkaido, Japan; kjpnplm0902@gmail.com (K.U.); tennisproyg@hotmail.com (Y.G.); nanakoushio75@gmail.com (N.U.); ragabfereig2018@gmail.com (R.M.F.); fumiaki.ihara@icloud.com (F.I.); 2 Laboratory of Veterinary Pathology, Department of Veterinary Medicine, Obihiro University of Agriculture and Veterinary Medicine, Obihiro 080-8555, Hokkaido, Japan; knabe@obihiro.ac.jp; 3 Department of Animal Medicine, Faculty of Veterinary Medicine, South Valley University, Qena City 83523, Qena, Egypt; 4 Division of Animal Science, Department of Agricultural and Life Sciences, Faculty of Agriculture, Shinshu University, Minamiminowa 399-4598, Nagano, Japan; tanakasa@shinshu-u.ac.jp; 5 Graduate School of Frontier Science, The University of Tokyo, Kashiwa 277-8561, Chiba, Japan; ysuzuki@k.u-tokyo.ac.jp

**Keywords:** *Toxoplasma gondii*, CXCR3, transcriptome, brain, astrocyte, microglia

## Abstract

The obligate intracellular parasite *Toxoplasma gondii* infects warm-blooded animals, including humans. We previously revealed through a whole-brain transcriptome analysis that infection with *T. gondii* in mice causes immune response-associated genes to be upregulated, for instance, chemokines and chemokine receptors such as CXC chemokine receptor 3 (CXCR3) and its ligand CXC chemokine ligand 10 (CXCL10). Here, we describe the effect of CXCR3 on responses against *T. gondii* infection in the mouse brain. In vivo assays using CXCR3-deficient mice showed that the absence of CXCR3 delayed the normal recovery of body weight and increased the brain parasite burden, suggesting that CXCR3 plays a role in the control of pathology in the brain, the site where chronic infection occurs. Therefore, to further analyze the function of CXCR3 in the brain, we profiled the gene expression patterns of primary astrocytes and microglia by RNA sequencing and subsequent analyses. CXCR3 deficiency impaired the normal upregulation of immune-related genes during *T. gondii* infection, in astrocytes and microglia alike. Collectively, our results suggest that the immune-related genes upregulated by CXCR3 perform a particular role in controlling pathology when the host is chronically infected with *T. gondii* in the brain.

## 1. Introduction

*Toxoplasma gondii*, an obligate intracellular parasite, infects a wide range of animals, including humans. The parasite has two different stages in its intermediate hosts: tachyzoites and bradyzoites. Tachyzoites are the fast-replicating parasite stage that occurs during acute infections, whereas bradyzoites are the slowly multiplying encysted stage present in host tissues during chronic latent infections. A major source of human infections arises from bradyzoite contamination of meat [1], and a third of the world’s population is reportedly chronically infected with this parasite [2]. Toxoplasmosis is generally asymptomatic in immunocompetent humans, but it also causes severe clinical diseases in immunocompromised individuals and pregnant mothers and infants with congenital disorders [3]. Uncontrolled parasite replication (called toxoplasmic encephalitis) causes life-threatening brain damage, as characterized by brain abscesses and necrotic brain areas. Toxoplasmic encephalitis is one of the primary causes of death in patients with human immunodeficiency virus/acquired immunodeficiency syndrome [4]. Primary infections with *T. gondii* in pregnant women can cause hydrocephalus or developmental disorders in the developing fetus [2].

Although *T. gondii* is capable of infecting most nucleated cells in vitro [5], acute infections with it also stimulate immune responses in the host. Interferon-gamma (IFN-γ) signaling and subsequent activation of nuclear factor-kappa B (NF-κB) are central immune responses against the parasite, and such responses result in high proinflammatory cytokine levels, such as interleukin (IL)-12 and IL-6 [6]. Existing evidence supports a role for *T. gondii* in inhibition and also activation of the NF-κB pathway in host cells [7,8]. Although the resultant immune attack and stressed condition inactivates *T. gondii* in most parts of the body, infected immune cells concurrently bring the parasite to the sites of latent infection, including the brain [9,10].

The human brain’s cell population is composed of glial cells, such as astrocytes and microglia, as well as neurons. Although glial cells can also become infected with *T. gondii* tachyzoites, previous studies have suggested that neurons are the primary targets of this parasite because the in vivo parasite infection is primarily found in neurons during the chronic infection phase [11,12]. Glial cells are considered to be responsible for immune responses that reduce neuronal and brain function damage, but their precise role in *T. gondii* infection is complicated to unravel. Astrocytes are the most abundant cell type in the brain, and they participate in many brain activities, including brain development and neurotransmission [13,14]. Astrocytes are efficiently infected by *T. gondii* but also seem able to activate protective immune responses within the central nervous system (CNS) [15,16]. Microglia are the resident macrophages in the brain and are important cellular effectors of neuroinflammation during brain injury and disease [17,18]. Although they trigger anti-parasite immunity in *T. gondii* infections and diminish parasite replication, they may also act as “Trojan horses”, thereby expanding the infection [11,19].

Previously, our group profiled gene expression in the whole mouse brain after 32 days of infection with *T. gondii*, and found that expression of CXC chemokine receptor 3 (CXCR3) and its ligand, CXC chemokine ligand 10 (CXCL10), was upregulated by the infection along with other immune response-associated genes [20]. Chemokines are one of many important factors involved in protection against a *T. gondii* infection, because their function promotes the migration of immune cells to the sites of infection. CXCR3 and its ligands (i.e., CXCL9, CXCL10, and CXCL11) have been reported to be associated with the Th1 immune response via the control of T cell migration [21,22,23,24,25]. In intestinal infections with *T. gondii*, the absence of CXCR3 impairs the recruitment of CD4⁺ T cells and the secretion of IFN-γ, resulting in the loss of protective immunity caused by the impaired activation of inflammatory monocytes [26]. CXCL10 (IP-10) is known to mediate T cell migration and has been reported to be critical for host survival in *T. gondii* infections [27]. CXCL10 is reported to be essential for the maintenance of T cell populations and for the control of parasite replication during chronic ocular toxoplasmosis [28]. CXCL10 has also been found to enhance the ability of CD8^+^ T cells to control *T. gondii* infection in the brains of chronically infected mice by maintaining the brain’s effector T cell population and increasing this population’s migration speed [29]. CXCR3 is also expressed on many other cell types, including macrophages, astrocytes, microglia, and neurons [30,31]. The chemokine system participates in neuroinflammatory processes and the development and functioning of the CNS, which includes neuron–glia communication and neuroendocrine activity [32]. However, whether an association exists between the immune responses occurring during *T. gondii* infection and the role of CXCR3 in the brain is unclear.

In this study, we conducted in vivo challenge experiments in the presence or absence of CXCR3 to examine the effect of CXCR3 on the control of *T. gondii* infections in mice. Our results showed that body weight recovery slowed down in the absence of CXCR3 during the sub-acute to chronic infection period. Using RNA-sequencing (RNA-seq) approaches, we examined the transcriptome profiles of in vitro cultured primary astrocytes and microglia to overview the function of CXCR3 in the brain. The normal upregulation of the genes associated with immune responses during *T. gondii* infection was impaired by CXCR3 deficiency, and the gene expression patterns were consistent with the results from our other quantitative assays. By elucidating the role of CXCR3 in glial cell-related immune response triggering against *T. gondii*, our findings advance current understanding of the mechanism controlling *T. gondii* infection in the brain.

## 2. Materials and Methods

### 2.1. Ethics Statement

Our study was performed in strict accordance with recommendations of the Guide for the Care and Use of Laboratory Animals of the Ministry of Education, Culture, Sports, Science and Technology, Japan. The protocol was approved by the Committee on the Ethics of Animal Experiments at Obihiro University of Agriculture and Veterinary Medicine, Hokkaido, Japan (permit number 19-51, 19-189). All surgeries were performed under isoflurane anesthesia with every effort made to minimize animal suffering.

### 2.2. Animals

C57BL/6J (WT) mice were purchased from Clea Japan (Tokyo, Japan). CXCR3KO mice (B6.129P2-Cxcr3tm1Dgen/J, Stock Number: 005796) were purchased from the Jackson Laboratory (Bar Harbor, ME). The CXCR3KO mice have been backcrossed to C57BL/6 at least six times before being imported to our laboratory. All animals were housed in cages (<6 mice/cage, 225 mm × 340 mm × 155 mm) containing wood chip bedding under specific-pathogen-free conditions in the animal facility of the National Research Center for Protozoan Diseases at Obihiro University of Agriculture and Veterinary Medicine. Male mice at 9–11 weeks old were used for in vivo experimental infection and preparation of peritoneal macrophages. The mean ± SD starting weight was 23.6 ± 1.0 g for WT mice and 23.7 ± 1.3 g for CXCR3KO mice. Mice at 6–8 weeks old were used as the parents of fetal mice for the preparation of primary glial cells.

### 2.3. Parasites

*T. gondii* tachyzoites (PLK strain, type II) were serially passaged in Vero cell monolayers. The parasites and cells were maintained in minimum essential medium (Sigma–Aldrich, St. Louis, MO) supplemented with 8% (*v/v*) fetal bovine serum (FBS; Biowest, Nuaillé, France) and 1× penicillin–streptomycin (PS; FUJIFILM Wako Pure Chemical Corporation, Osaka, Japan) at 37 °C in humidified air with 5% CO_2_. To purify the parasites, cell monolayers were scraped, centrifuged, and then resuspended in the culture medium for each assay. Aggregated tachyzoites and host cell debris were removed by repeatedly passing the suspension through a 27-gauge needle and filtration through a 5.0-µm pore-size filter (Millipore, Burlington, MA, USA).

### 2.4. In Vivo Infection and Sample Collection

WT and CXCR3KO male mice (*n* = 6 per group) were intraperitoneally injected with 1000 and 10,000 tachyzoites suspended in 400 µL of Roswell Park Memorial Institute (RPMI)-1640 medium (Sigma–Aldrich). Survival and body weight of the mice were monitored daily throughout the experimental period. The hair coat, posture, and behavior of the mice were also monitored during the experiments and were scored on a scaled of 0 to 4. At 60 days post infection (dpi); surviving mice were anesthetized and subjected to blood collection by cardiac puncture using a 23 G needle and 1 mL syringe. Mice were then sacrificed by cervical dislocation and the brain was removed and cut into two hemispheres. The left brain was fixed in 10% formalin neutral buffer solution (FUJIFILM Wako Pure Chemical Corporation) for histological analysis. The right brain was stored at −30 °C until DNA extraction. The serum of each mouse was tested by an enzyme-linked immunosorbent assay (ELISA) against *T. gondii* dense granule protein 7 to confirm the infection following our previously described method [33].

### 2.5. Quantification of Tissue Parasites 

For DNA extraction, tissue samples were lysed in a lysis buffer (1% SDS, 0.1 M Tris HCl [pH8.0], 0.1 M NaCl, 1 mM EDTA [pH8.0]) and DNA was purified by Phenol:Chloroform:Isoamyl Alcohol (25:24:1) (Nakarai Tesque, Inc., Kyoto, Japan) and ethanol precipitation. Extracted DNA was subjected to real-time PCR using primer pairs specific for B1 gene of *T. gondii* (Forward 5’-AAC GGG CGA GTA GCA CCT GAG GAG-3’ and Reverse 5’-TGG GTC TAC GTC GAT GGC ATG ACA AC-3’). PCR reactions were performed in 10 µL of reaction volume containing 1× PowerUp SYBR Green Master mix (Applied Biosystems, Waltham, MA), 500 nM each primer, and 50 ng of genomic DNA in an ABI Prism 7900HT sequence detection system (Applied Biosystems). Thermal cycling conditions were as follows: 2 min at 50 °C, 10 min at 95 °C, 40 cycles at 95 °C for 15 s, and 60 °C for 1 min, followed by a dissociation step from 60 °C to 95 °C to confirm gene-specific amplification. The number of parasites per 50 ng of tissue DNA was calculated from a standard curve, which was established from serially diluted DNA extracted from purified tachyzoites (0.01 to 10,000 parasites per reaction).

### 2.6. Histological Analysis

Formalin-fixed brain tissue was embedded in paraffin and sectioned at 4 µm. Six levels of brains (frontal lobe, thalamus, striatum, occipital lobe, brain stem, and cerebellum) were examined. The sections were subjected to hematoxylin–eosin staining or immunohistochemical staining using rabbit polyclonal antibodies against *Toxoplasma gondii* (Quartett Immunodiagnostica, Berlin, Germany), glial fibrillary acidic protein (GFAP) (Thermo Fisher Scientific, Waltham, MA, USA), IBA1 (FUJIFILM Wako Pure Chemical Corporation) and CD4 (Bioss Antibodies Inc., Woburn, MA, USA) after antigen retrieval at 98 °C for 45 min with immunosaver (Nisshin EM Co., Ltd, Tokyo, Japan) and using a rat monoclonal antibody against CD8a (Thermo Fisher Scientific, clone: 4SM15) after antigen retrieval at 98 °C for 15 min in citrate buffer (pH 6.0) with microwave oven. For quantification analysis of astrocytes and microglia activation, area positive for GFAP and IBA1 were assessed because astrocytes and microglia were increased in the number, thickness, and length of the main cellular processes not only the numbers of the cells by the activation. For quantification analysis of T cell population, the numbers of cells positive for CD8a and CD4 were assessed. In detail, 10 images of the cerebral cortex were randomly taken at ×100 magnification in each mouse. Areas positive for GFAP or IBA1 were quantified using Adobe Photoshop (Adobe Systems Co., Ltd., Tokyo, Japan) and ImageJ [34]. The number of cells positive for CD8a and CD4 were counted in the 10 images. *T. gondii* tissue cysts were counted in 10 images (×100 magnification) of the cortex, hippocampus, caudoputamen, amygdala, thalamus, hypothalamus, midbrain, and cerebellum.

### 2.7. In Vitro Preparation of Primary Murine Cells

#### 2.7.1. Astrocytes

Astrocytes were obtained from the brain cortex of fetal mice (age, E17–18) according to a previously described procedure [35,36], with some modifications. After removal of the meninges from the fetal brain, the cortex was mechanically dissociated in Dulbecco’s modified Eagle medium (DMEM; Sigma–Aldrich) containing 0.25% trypsin and 0.01% DNase to obtain a single-cell suspension and was incubated at 37 °C for 10 min. The dissociated cells were washed and suspended in DMEM/F-12 (Gibco-BRL, Grand Island, NY) supplemented with 10% FBS, 1× PS, and 1× G-5 Supplement (Gibco-BRL). The cells were plated in 75-cm^2^ flasks (density, 2 × 10^6^ cells/flask) and were then incubated at 37 °C. The culture medium was changed every 3 days until the culture reached confluence. After 7–8 days of incubation, the astrocyte monolayers were washed with medium and were dissociated with 0.25% trypsin–EDTA solution. The dissociated astrocytes were centrifuged at 500× *g* for 5 min at 4 °C and were washed in DMEM/F-12 supplemented with FBS, PS and G-5 Supplement. Then, 2 × 10^5^ cells in 500 µL medium were reseeded in each well of 24-well plates and were allowed to grow for 16 h before being infected.

#### 2.7.2. Microglia

Microglial cells were obtained using a procedure similar to that used for astrocytes, with some modifications [35]. Dissociated brain cells were washed and suspended in DMEM/F-12 supplemented with 10% FBS, 1× PS and 10 ng/mL of granulocyte-macrophage colony-stimulating factor (R&D Systems, Minneapolis, MN, USA). The cells were plated in 75-cm^2^ flasks (density, 4 × 10^6^ cells/flask) and the culture medium was changed every 3 days. After 10–11 days of incubation, the microglial cells were detached from the astrocyte monolayer by pipetting and were centrifuged at 500× *g* for 5 min at 4 °C. Then, 2 × 10^5^ cells in 500 µL medium were reseeded in each well of 24-well plates and were allowed to grow for 16 h before being infected.

#### 2.7.3. Peritoneal Macrophages

Peritoneal macrophages were isolated from peritoneal cavities 4 days after injection of 1 mL of 4.05% thioglycollate medium according to a previously described procedure [35]. Peritoneal exudate cells were harvested by lavage with 5 mL of cold phosphate-buffered saline (PBS) and filtered through a 40-μm cell strainer to remove cell aggregates and debris. After centrifugation at 1000× *g* for 5 min, pelleted cells were resuspended in DMEM supplemented with 10% FBS and 1× PS, and 5 × 10^5^ cells in 500 µL medium were seeded in each well of 24-well plates and were allowed to attach for 20 h. The macrophages were then washed twice with DMEM to remove the non-adherent cells. 

### 2.8. RNA-Seq Analysis

Astrocytes and microglia were infected with *T. gondii* tachyzoites for 24 h at a multiplicity of infection (MOI) of 1.0. Infected and uninfected groups of cells were prepared in triplicate wells for each mouse genotype and each cell type. Total RNA was extracted with TRI Reagent^®^ (Sigma–Aldrich), according to the manufacturer’s protocol. The extracted RNA was individually subjected to RNA-seq, according to the protocol used in our previous study [20,35,37]. Briefly, 1 µg of total RNA was subjected to poly-A selection. Sequencing libraries were constructed using the TruSeq RNA sample preparation kit (Illumina, San Diego, CA), while 36-bp single-end sequencing was performed using the Illumina Genome Analyzer IIx and TruSeq SBS Kit v5-GA (36-cycle) (Illumina), according to the manufacturer’s instructions. All treatments and subsequent analyses were performed on individual transcripts. The data of WT samples has been used in our previously published articles [35,37] because all the samples in these articles and the present study were prepared simultaneously. Raw sequence reads were subjected to quality control, and the cleaned reads were mapped to the reference mouse genome (mm10) with CLC Genomics Workbench version 10 (CLC bio, Aarhus, Denmark) (read mapping parameters: minimum fraction length of read overlap = 0.95, minimum sequence similarity = 0.95). 

### 2.9. Identification of Differentially Expressed Genes (DEGs) in Which Upregulation during T. Gondii Infection Was Impaired by CXCR3-Deficiency

Based on the mapping results, differential expression analysis was performed for pairwise comparisons of the expression data using the R packages DESeq2 [38] and edgeR [39,40]. In this study, only genes that appeared differentially expressed in both DESeq2 and edgeR were considered as DEGs. DEGs in which upregulation during infection was impaired by CXCR3-deficiency were defined as follows: (1) log2 fold-change >1 and false discovery rate (FDR) < 0.05 between the infected and uninfected WT mice; (2) FDR < 0.05 between the infected WT and the infected CXCR3KO mice; (3) the fold-change was higher in “uninfected WT vs. infected WT” than in “uninfected KO vs. infected KO”. We also identified genes up/downregulated just by CXCR3-deficiency regardless of *T. gondii* infection (Table S1). For comparison among four groups (i.e., uninfected WT, infected WT, uninfected CXCR3KO, and infected CXCR3KO), the raw-read counts for each gene were normalized by the iDEGES method implemented in the TCC package [41].

### 2.10. Functional Enrichment Analyses of DEGs in Which Upregulation during T. gondii Infection Was Impaired by CXCR3-Deficiency

The DEGs in which upregulation during *T. gondii* infection was impaired by CXCR3-deficiency were functionally categorized by gene ontology (GO) term enrichment analysis. Statistical overrepresentation of GO terms for selected genes was compared with the reference genes (all genes; 37,315 genes) using the goseq package in the R [42]. Genome-wide annotation for the mouse was obtained using the org.Mm.eg.db package [43]. Functional annotation charts for the enriched GO terms were generated using the GO terms associated with biological processes. Only GO terms with FDRs of <0.05 and the number of DEGs ≥ 10 were considered to represent functional enrichment.

The Kyoto Encyclopedia of Genes and Genomes (KEGG) database is a bioinformatics tool that assembles large-scale molecular datasets, such as gene lists, into biological pathway maps [44]. We performed KEGG pathway enrichment analysis on the DEG list using the clusterProfiler package [45] in the R to assess the overarching functions of the DEGs in which upregulation during *T. gondii* infection was impaired by CXCR3-deficiency. 

### 2.11. Cytokine ELISA

Using cell populations different from those used for RNA-seq, astrocytes, microglia, and macrophages were infected with *T. gondii* tachyzoites for 24 h. The MOIs were 1.0 for astrocytes and microglia and 0.2 for macrophages. We used a lower MOI for macrophages because many macrophages infected at MOI of 1.0 were detached from the well surface and seemed to be dying in our preliminary experiment. Infected and uninfected groups of cells were prepared in 4 replicate wells for each mouse genotype. The culture supernatant was collected and subjected to cytokine ELISA for IL-6 and IL-12p40 using corresponding BD OptEIA™ ELISA kits (BD Bioscience, San Jose, CA) according to the manufacturer’s protocol. The supernatants were also subjected to a nitrite/nitrate assay using a nitrite/nitrate assay kit (Cayman Chemical Co., Ann Arbor, MI), according to the manufacturer’s instruction. The nitrite and nitrate levels were calculated with a standard absorbance curve constructed from samples run on the same plate. 

### 2.12. Reverse Transcription-Quantitative Polymerase Chain Reaction (RT-qPCR)

After the collection of the culture supernatant for cytokine ELISA, total RNA was extracted from astrocytes and microglia with TRI Reagent^®^. cDNA was synthesized from 400 ng of each RNA sample using PrimeScript™ RT Master Mix (Perfect Real Time) (Takara Bio Inc., Shiga, Japan) following the manufacturer’s instruction. PCR parameters used were the same as described above. Primers used were listed in Table S2. The cycle threshold (C_t_) values were normalized to the expression levels using the 2^−ΔΔCt^ method [37,46]. *Gapdh* was used as the internal control gene after comparison with *Actb* using RefFinder [47]. The calibrator sample was the uninfected WT group.

### 2.13. Statistical Analysis

Survival curves were compared with the log-rank test. Changes in body weight were compared with repeated-measures two-way ANOVA. The number of parasites in the brain was compared by two-tailed unpaired Student’s t-test. Areas positive for astrocyte and microglia markers in the brain sections were compared between genotypes by two-tailed unpaired Student’s t-test. The numbers of cells positive for CD8a and CD4 were compared between genotypes by two-tailed unpaired Student’s t-test. The results of cytokine ELISA and RT-qPCR were compared by two-way ANOVA with post-hoc Tukey’s test among groups.

## 3. Results

### 3.1. In Vivo Effects of CXCR3-Deficiency during T. gondii Infection

**Figure 1:**
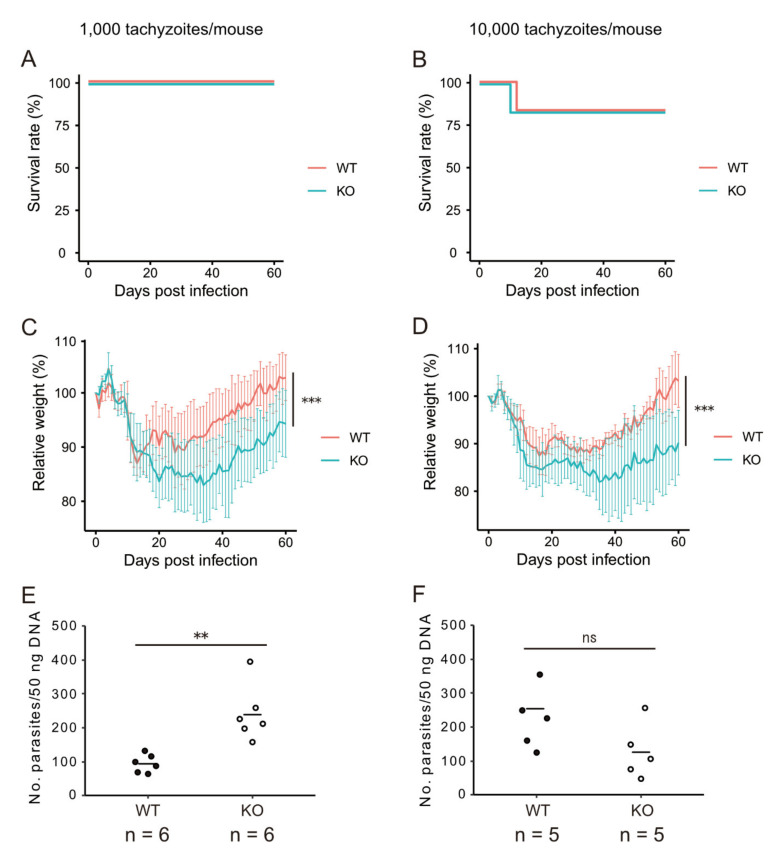
Survival, body weight changes and brain parasite burden in WT and CXCR3KO mice infected with *T. gondii*. WT and CXCR3KO mice (*n* = 6 per group) were intraperitoneally injected with 1000 (**A**,**C**,**E**) or 10,000 (**B**,**D**,**F**) tachyzoites per mouse. (**A**,**B**) Survival rate. (**C**,**D**) Relative body weights based on the weights at the beginning of the experiment. Lines and error bars represent mean ± SD. *** *p* < 0.001 for the interaction between mouse genotype and days post infection (two-way ANOVA). (**E**,**F**) Number of parasites per 50 ng of DNA extracted from brain tissue from each surviving mouse at 60 dpi. Horizontal bars represent the mean value. ns, *p* > 0.05; ** *p* < 0.01, two-tailed unpaired Student’s *t*-test.

We first investigated the effect of CXCR3-deficiency using in vivo challenge experiments. WT and CXCR3KO mice (*n* = 6 per group) were intraperitoneally injected with 1000 or 10,000 tachyzoites (PLK strain, type II) and monitored daily. No significant differences were observed for the survival of the infected mice between the two genotypes (log-rank test, *p* > 0.05) (Figure 1A,B). However, body weight changes in the mice showed a significant interaction between mouse genotype and dpi for both infection doses (two-way ANOVA, *p* < 0.001) (Figure 1C,D). Poor condition of the hair coat continued for a longer period in CXCR3KO mice than in WT mice although difference in the other clinical signs was not as clear between genotypes (Figure S1). Two other experiments repeated under similar conditions showed the same relative body weight trends. We also investigated the parasite burden in the brain tissues from the surviving mice at 60 dpi. For mice injected with 1000 tachyzoites, the parasite burden was significantly higher in the CXCR3KO mice than in the WT mice (two-tailed unpaired Student’s *t*-test, *p* < 0.01), while there was no significant difference (*p* > 0.05) for mice injected with 10,000 tachyzoites (Figure 1E,F).

**Figure 2:**
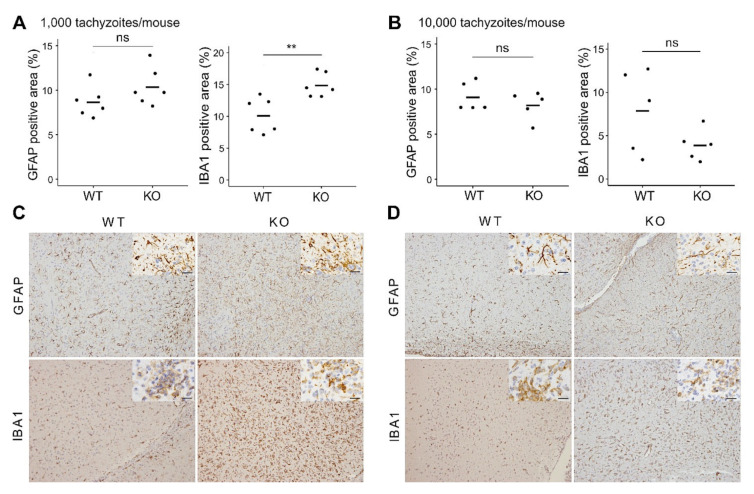
Astrocyte and microglia populations in the frontal lobes of *T. gondii*-infected mice. The brains from WT and CXCR3KO mice surviving at 60 dpi were subjected to histopathological processing. Astrocyte and microglia populations were identified using immunohistochemical staining against glial fibrillary acidic protein (GFAP) and ionized calcium-binding adapter molecule 1 (IBA1), respectively. (**A**,**B**) Positive areas were compared between WT and CXCR3KO. Horizontal bars represent the mean value. ns, *p* > 0.05; ** *p* < 0.01, two-tailed unpaired Student’s *t*-test. (**C**,**D**) Representative immunohistochemical images were taken from serial tissue sections containing both astrocytes and microglia. (**A**,**C**) 1000 tachyzoites per mouse (*n* = 6 per group). (**B**,**D**) 10,000 tachyzoites per mouse (*n* = 5 per group). Magnified images were shown in the top right of each picture of astrocytes and microglia. Scale bars, 20 µm.

To further analyze brain inflammation in the mice, the brains from WT and CXCR3KO mice surviving at 60 dpi were subjected to histopathological analysis. Nonsuppurative meningoencephalitis, perivascular cuffing, and glial cell proliferation were diffusely observed in all groups (Figure S2A–C). Parasite cells were immunohistochemically identified with an anti-*T. gondii* antibody. The parasite had encysted itself in all regions of the examined brains, and few tachyzoites were observed in inflammatory lesions (Figure S2D–F). Although some cysts were distributed in the cortex, their numbers were too low and insufficient to allow a comparison to be made between the WT and CXCR3KO mice (Figure S3). Cell-type specific immunohistochemistry was also performed, and the two genotypes were compared. While the astrocyte populations showed no significant differences in the areas positive for GFAP (two-tailed unpaired Student’s *t*-test, *p* > 0.05), microglia populations (IBA1 positive) in the brains of mice injected with 1000 tachyzoites were significantly higher in the CXCR3KO mice than in the WT mice (*p* < 0.01) (Figure 2). While the number of CD4^+^ T cell showed no significant differences, the number of CD8a^+^ T cell in the brains of mice injected with 10,000 tachyzoites were significantly higher in the WT mice than in the CXCR3KO mice (*p* < 0.05, two-tailed unpaired Student’s t-test) (Figure S4).

### 3.2. Effects of CXCR3-Deficiency on Gene Expression in Primary Glial Cells during T. gondii Infection

We next investigated the gene expression patterns in primary glial cells using RNA-seq approaches. RNA-seq and differential expression analysis were carried out on primary astrocytes and microglia infected with *T. gondii* for 24 h at a MOI of 1.0.

**Figure 3:**
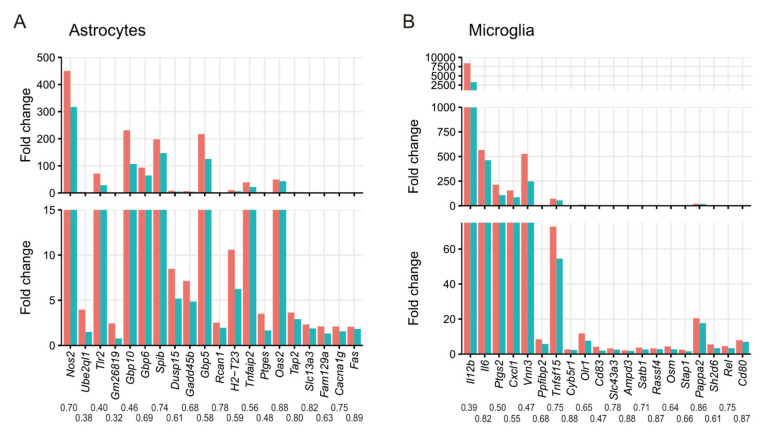
Expression patterns of the top 20 DEGs whose upregulation during *T. gondii* infection was impaired by CXCR3-deficiency. DEGs were ranked according to the fold changes between the infected WT vs. infected CXCR3KO mice. (**A**) Astrocytes. (**B**) Microglia. Each bar represents the fold change between uninfected vs. infected mice. Red, uninfected WT vs. infected WT; green, uninfected CXCR3KO vs. infected CXCR3KO. Each value under a gene symbol represents the ratio of the fold-change between uninfected KO vs. infected KO to that between uninfected WT vs. infected WT. The area of low fold-change is enlarged in each panel.

Our differential expression analysis on astrocytes identified 79 genes whose upregulation during *T. gondii* infection was impaired by CXCR3-deficiency (Table S3). The functions of these DEGs were assessed by GO term enrichment analysis (Table 1 and Table S4). Among the genes whose upregulation during infection was impaired by CXCR3-deficiency, overrepresented GO terms were associated with immune responses and categories associated with stress responses. Our KEGG pathway enrichment analysis also showed that infectious disease-related pathways were activated via CXCR3 during *T. gondii* infection (Table S5). To investigate in detail how the absence of CXCR3 affects astrocytes, DEGs, whose upregulation during infection was impaired by CXCR3-deficiency, were ranked according to the FDR between *T. gondii*-infected cells from WT and CXCR3KO mice (Figure 3A). Most of the top 20 genes were IFN-γ inducible genes (i.e., *Nos2*, *Tlr2*, *Gbp10*, *Gbp6*, *Gadd45b*, *Gbp5*, *Rcan1*, *H2-T23*, *Tnfaip2*, *Ptges*, *Oas2*, *Tap2*, *Slc13a3*, *Fam129a*, and *Fas*). NF-κB target genes (i.e., *Nos2*, *Tlr2*, *Gadd45β*, *Tnfaip2*, *Ptges*, and *Fas*) were also abundant in the top-ranking genes.

Our differential expression analysis on microglia identified 86 genes that were upregulated during *T. gondii* infection but were impaired by CXCR3-deficiency (Table S3). The functions of these DEGs were assessed by GO term enrichment analysis (Table 1 and Table S4). Among the genes whose upregulation during infection was impaired by CXCR3-deficiency, overrepresented GO terms were associated with inflammatory responses and cytokine production. In the KEGG pathway enrichment analysis, cytokine–cytokine receptor interactions were overrepresented, as were infectious disease-related pathways (Table S5). The identified DEGs were ranked in the same way as described above, and the expression levels of the top 20 genes were compared (Figure 3B). Most of the top 20 genes were IFN-γ inducible genes (i.e., *Il12b*, *Il6*, *Ptgs2*, *Cxcl1*, *Tnfsf15*, *Olr1*, *Cd83*, *Slc43a3*, *Ampd3*, *Satb1*, *Rassf4*, *Osm*, *Stap1*, and *Rel*) and NF-κB target genes (i.e., *Il12b*, *Il6*, *Ptgs2*, *Cxcl1*, *Tnfsf15*, *Olr1*, *Cd83*, *Rel*, and *Cd80*).

In this study, as we aimed to investigate the effect of CXCR3 on the expression characteristics of genes during *T. gondii* infection, we focused on DEGs whose upregulation during *T. gondii* infection was impaired by CXCR3-deficiency. Some genes were regulated upwards or downwards simply because of CXCR3-deficiency, regardless of an active *T. gondii* infection (Table S1). Among these, *Ftl1* and *Ftl1-ps1* were ranked as the most upregulated and downregulated, respectively, in both astrocytes and microglia. However, this finding is likely related to a limitation in the software algorithm because these two genes are >99.3% homologous within their overlaps.

**Figure 4:**
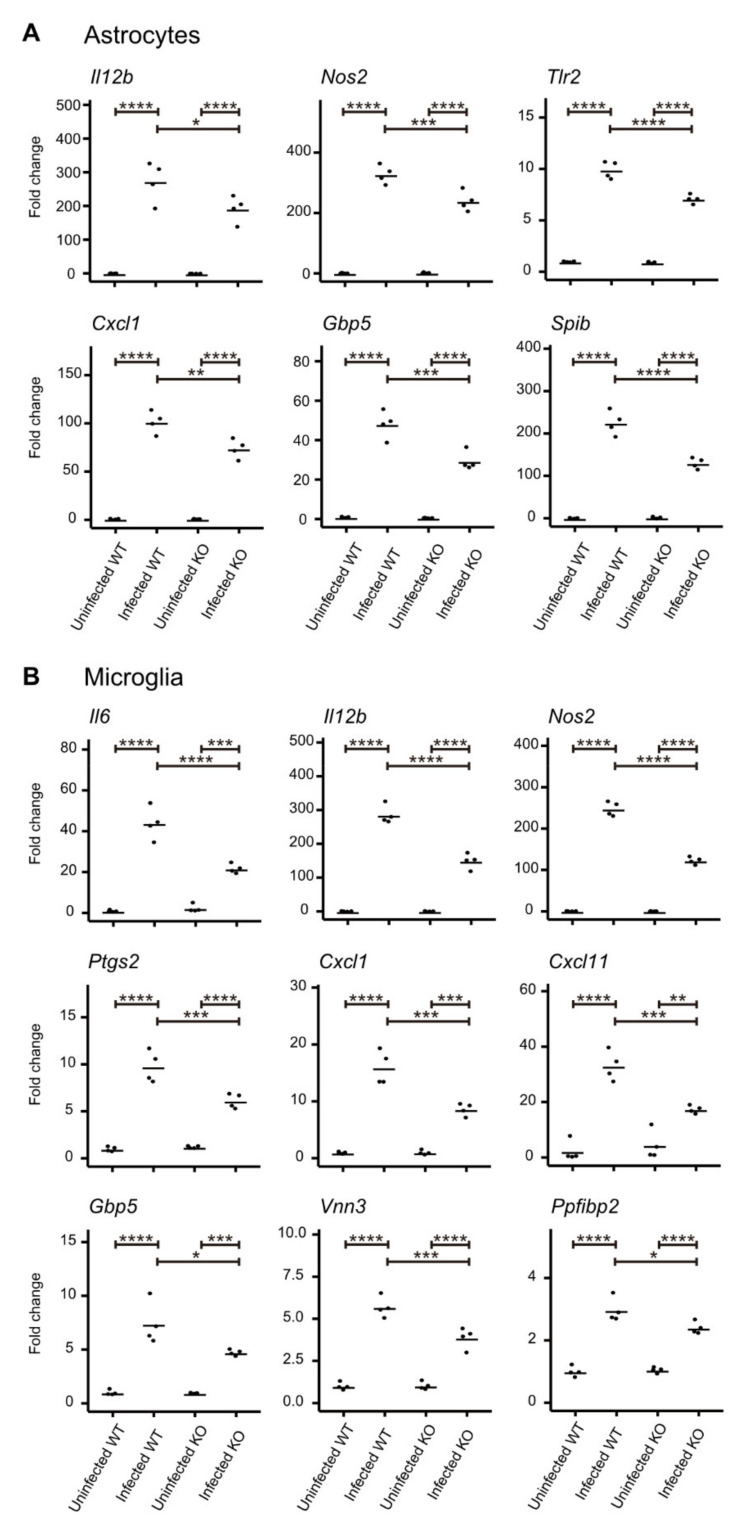
Expression patterns of DEGs in primary astrocytes and microglia during *T. gondii* infection. Primary astrocytes (**A**) and microglia (**B**) were infected with *T. gondii* tachyzoites for 24 h at a MOI of 1.0. Genes with a significant interaction between genotype and infection (*p* < 0.05, two-way ANOVA) are shown. Horizontal bars represent the mean values of four replicate samples in each group. * *p* < 0.05, ** *p* < 0.01, *** *p* < 0.001, **** *p* < 0.0001 (two-way ANOVA with post-hoc Tukey’s test).

To confirm the expression patterns shown by RNA-seq, seven and nine genes were selected from the top-ranking genes shown in Figure 3 for astrocytes (*Dusp15, Gadd45b*, *Gbp5*, *Nos2*, *Tlr2*, *Spib*, and *Ube2ql1*) and microglia (*Cd83*, *Cyb5r1*, *Cxcl1*, *Il6*, *Il12b*, *Ptgs2*, *Ppfibp2*, *Tnfs15,* and *Vnn3*), respectively, along with genes encoding CXCR3 ligands (*Cxcl9*, *Cxcl10*, and *Cxcl11*). The expression profiles of these genes were examined by RT-qPCR analysis. The two-way ANOVA and post-hoc Tukey’s test showed that the lack of CXCR3 impaired the upregulation of *Il12b*, *Nos2*, *Tlr2*, *Cxcl1*, *Gbp5* and *Spib* in astrocytes (Figure 4A) and *Il6*, *IL12b*, *Nos2*, *Ptgs2*, *Cxcl1*, *Cxcl11*, *Gbp5*, *Vnn3* and *Ppfibp2* in microglia (Figure 4B). No interactions were observed between mouse genotype and *T. gondii* infection for the expression of other genes (Figure S5).

### 3.3. In Vitro Cytokine Production from Primary Glial Cells and Macrophages

**Figure 5:**
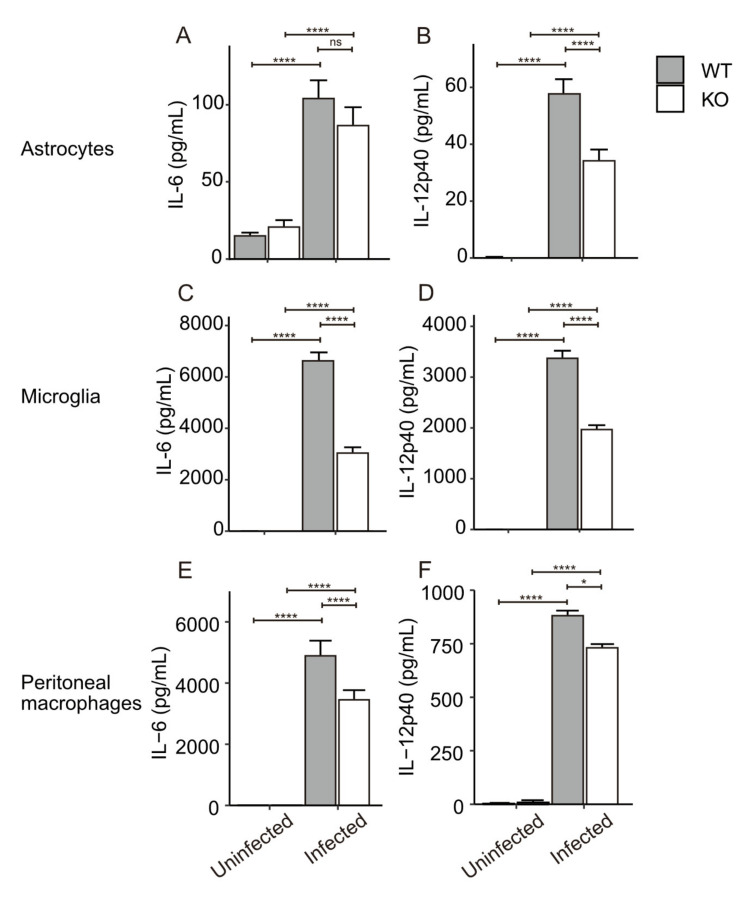
Cytokine production from primary astrocytes, microglia and macrophages during *T. gondii* infections. Primary astrocytes (**A**,**B**), microglia (**C**,**D**), macrophages (**E**,**F**) were infected with *T. gondii* tachyzoites for 24 h at MOIs of 1.0, 1.0, and 0.2, respectively. Cells were prepared in four replicate wells for each group. Bars and error bars represent the mean ± SD. ns, *p* > 0.05, * *p* < 0.05, **** *p* < 0.0001 (two-way ANOVA with post-hoc Tukey’s test). These experiments were repeated twice and the representative data were presented.

IL-6 and IL-12 are well-known inflammatory cytokines. As shown above, their mRNA expression was affected by CXCR3-deficiency during *T. gondii* infection. To confirm the effect on their protein expression, cytokine productions from CXCR3-deficient glial cells were also examined using a cytokine ELISA against IL-6 and IL-12p40. IL-12p40 production from the infected astrocytes was significantly lower (by 40%) in the CXCR3KO mice than in the WT mice, whereas IL-6 production did not significantly differ between the two genotypes (Figure 5A,B). However, the concentrations of both of these cytokines during the infection were much lower in astrocytes than in microglia. IL-6 and IL-12p40 production from microglia were both highly upregulated during infection with *T. gondii*, but CXCR3 deficiency significantly decreased the concentrations of these cytokines by 54% and 42%, respectively (Figure 5C,D). Additionally, we examined thioglycollate-elicited peritoneal macrophages because these cells have important roles in peripheral immune responses. Peritoneal macrophages, which we infected with *T. gondii* in vitro at a MOI of 0.2, were subjected to cytokine ELISA. IL-6 and IL-12p40 production markedly increased in both WT and CXCR3KO mice (Figure 5E,F). IL-6 and IL-12p40 productions were significantly lower (by 30% and 14%, respectively) in CXCR3KO mice than in WT mice. We also examined NO production from astrocytes and microglia during *T. gondii* infection, but no NO production was observed in these cells (data not shown).

## 4. Discussion

The CNS is the primary site of latent infection for *T. gondii*. Recent studies have reported that chemokine–chemokine receptor signaling plays roles in immune responses against *T. gondii* infection, not only in the peripheral immune system, but also in the CNS [35,37,48]. CXCR3 is known to perform important roles in the peripheral immune system. CXCR3 is associated with the recruitment of both CD4⁺ and CD8^+^ T cells to the site of infection, IFN-γ secretion from the T cells, and the resultant activation of inflammatory monocytes [26,49]. For example, CXCR3 activation can affect the proliferation and immune responses of macrophages and dendritic cells during infection with *Leishmania amazonensis* [50,51]. CXCR3 also controls the recruitment of microglia to the infection sites [52] and enhances proliferation of epithelial cells [53]. CXCR3 is constitutively expressed on neurons and neuronal processes, along with markedly elevated CXCL10 in astrocytes in the brains of people with Alzheimer’s disease [31]. However, the detailed role played by CXCR3 during *T. gondii* infection in the brain remains unclear.

Through our in vivo challenge experiments, we initially found that CXCR3 deficiency resulted in delayed body weight recovery and higher brain parasite burden in the mice, implying that this deficiency exacerbated clinical disease during the sub-acute (15–30 dpi) to chronic phases (30–60 dpi) of infection. We speculated that a high infection dose might result in a saturated immune response controlled by different pathways (e.g., Toll-like receptor (TLR) 2/MyD88 pathway) and this would hinder the effect of CXCR3 loss. Therefore, we utilized two different doses of parasites in the present study. This may explain why the difference in parasite burden and microglia frequency appeared to be more significant when the mice were infected with the lower dose of parasites. Survival rates did not differ between WT and CXCR3KO mice after intraperitoneal injection with type II *T. gondii* (PLK strain) tachyzoites. In contrast, in a previous study, CXCR3 deficiency resulted in lower mouse survival after oral inoculation of type II *T. gondii* ME49 strain cysts [26]. This difference is probably related to the different routes by which the experimental infections were initiated. In our present study, the infection should have spread more rapidly throughout its body, including to the brain, because *T. gondii* tachyzoites were directly injected into the host’s peritoneal cavity, as compared with the observations of our previous study. This might explain why we observed an exacerbation in the clinical signs of disease during the chronic infection phase. The immunohistochemical assay results suggest that the proliferation and/or migration of microglia in response to *T. gondii* infection might have been affected by the CXCR3 deficiency. Transcriptome analysis of the primary microglia revealed impaired expression of immune response-associated genes in the absence of CXCR3. Collectively, these results suggest that the lack of CXCR3 resulted in an impaired immune response in the brain at the earlier sub-acute stage and led to higher parasite burdens. This higher parasite burden might have triggered the increased population of microglia in the brain that we observed at 60 dpi. To learn more about the effect of CXCR3-deficiency on T cell recruitment to the brain during *T. gondii* infection, we examined the T cell population in the brain tissues from the surviving mice. We found a significant decrease in the number of CD8a^+^ T cells in CXCR3KO mice injected with 10,000 tachyzoites while no significant difference was observed in CD4^+^ T cells. This suggests that under our experimental conditions, the increased parasite burden in the brain might be associated with impaired CD8^+^ T cell recruitment. To further investigate brain cell populations during the infection, we turned our attention to brain glial cells and their gene expression profiles.

Using RNA-seq approaches, we profiled the gene expression patterns in primary brain glial cells exposed to *T. gondii* tachyzoites. Our differential expression analysis identified genes whose upregulation during *T. gondii* infection was impaired by CXCR3-deficiency. Functional enrichment analyses showed that in both astrocytes and microglia many of the genes were associated with immune and stress responses. RT-qPCR assays confirmed the expression patterns of the DEGs identified by RNA-seq, and the genes that showed the same patterns in both RNA-seq and RT-qPCR were *Nos2*, *Tlr2*, *Gbp5* and *Spib* for astrocytes and *Il6*, *Il12b*, *Ptgs2*, *Cxcl1*, *Vnn3* and *Ppfibp2* for microglia. Our mRNA-level observations were confirmed at the protein level using cytokine ELISAs. Production of IL-6 and IL-12p40 was lowered by CXCR3 deficiency, especially in microglia. We consider that the results of these in vitro experiments reflect the in vivo situation that occurred during the sub-acute phase of the infection, when *T. gondii* tachyzoites started their invasion from the peripheral body to occupy a more central position before they transformed into tissue cysts.

Nitric oxide (NO) production, which occurs through inducible nitric oxide synthase (iNOS) induction via the gene encoding *Nos2*, is an important microbicidal factor against *T. gondii* [54,55]. NO production was not found in astrocytes and microglia during *T. gondii* infection in the absence of IFN-γ, under the same conditions that we used for obtaining the transcriptome samples (data not shown). Although we have no data on NO production from these cells in the presence of IFN-γ, and it is not known whether CXCR3 affects NO production, NO production from astrocytes and microglia in the presence of IFN-γ reportedly decreases during type I *T. gondii* infection [56]. As well as the cytotoxic effects of NO, guanylate binding protein (GBP) genes, including *Gbp5*, are known to be important for controlling infection with *T. gondii*. GBPs play a critical role in destroying the parasitophorous vacuole membrane, along with interferon-inducible immunity-related GTPases [10,57]. *Gbp5*, an IFN-γ inducible gene in astrocytes, participates in activating the nucleotide-binding oligomerization domain-like receptor protein 3 inflammasome [16,58]. Because GBP5 has also been reported to be a marker for interferon-γ-induced classically activated macrophages [59], perhaps GBP5 is more susceptible to CXCR3 deficiency than other GBPs, although the detailed mechanism and function underlying its potential susceptibility is not known and therefore needs further investigation. A recent study reported that type III parasite clearage by naive murine macrophages depends on the enhanced activity of NADPH oxidase-generated reactive oxygen species and induction of *Gbp5* [60]. Toll-like receptor 2, encoded by *Tlr2*, is an important pattern recognition receptor for pathogen-associated molecular patterns and plays a critical role in mammalian innate immune responses [61,62]. Our previous study showed that TLR2 has a large impact on the expression of immune-related genes in murine brain cells [35]. *Spib* encodes the Spi-B transcription factor, which performs critical roles in plasmacytoid dendritic cell function and development [63]. However, the function of Spi-B in immune responses against *T. gondii* is unknown. Considering the functions of these genes, CXCR3 signaling plays a role in regulating anti-*Toxoplasma* activity in astrocytes.

IL-6 is known to have a protective role during early infection with *T. gondii* and it mediates susceptibility to the parasite [64,65], and IL-12, a well-known proinflammatory cytokine, stimulates IFN-γ synthesis by natural killer cells and T lymphocytes, and plays a critical role in resistance against *T. gondii* [66,67]. Another IFN-γ inducible gene, *Ptgs2*, encodes cyclooxygenase (COX)-2 and *T. gondii* is known to induce prostaglandin E2 synthesis in macrophages by inducing COX-2 [68]. A recent study showed that COX-2 inhibitors dampen down *T. gondii* infections and upregulate pro-inflammatory immune responses in rodent and human monocyte cell lines [69]. Microglia cells are also possibly involved in such interactions with *T. gondii* via this type of signaling. VNN3, also called vascular non-inflammatory molecule 3, is reportedly a potential prognostic biomarker for clear cell renal cell carcinoma [70], but no relationship with *T. gondii* infection has been reported thus far. Interestingly, *Ppfibp2*, another top-ranking gene, currently has an unknown function and unknown relationship with *T. gondii* infection. The DEGs listed above include many IFN-γ inducible genes despite the cultures not being supplemented with IFN-γ; hence, these genes should have been induced by infection alone. Low-level IFN-γ expression was observed in our transcriptome data (mean normalized counts were 0, 0, 0, and 0.41 in astrocytes and 0, 2.32, 0, and 3.41 in microglia for uninfected WT, infected WT, uninfected CXCR3KO, and infected CXCR3KO, respectively). The situation for IFN-β was that it was expressed to some extent although its expression level was still low and lacking any significant difference between the WT and KO mice (mean normalized counts were 0, 5.91, 0 and 3.63 in astrocytes, and 0.31, 11.1, 2.84, and 13.8 in microglia for the uninfected WT, infected WT, uninfected CXCR3KO, and infected CXCR3KO mice). IFN-β possibly impacted the expression of IFN inducible genes because the genes induced by these IFNs overlapped with each other [71,72,73].

The functions of the above-mentioned genes include both anti-*Toxoplasma* activity in host cells and host-regulating activity of the parasite, suggesting that microglia play a conflicted role during *T. gondii* infections. The expression of CXCR3 ligands was upregulated after *T. gondii* infection in vitro. After CXCR3 and its ligands interact, CXCR3 regulates downstream gene expression via p38 mitogen-activated protein kinase and PI3K signaling pathways [74,75,76,77]. These pathways possibly affect the production of inflammatory cytokines. Researchers have reported that TLR2/MyD88 activation has strong effects on the control of *T. gondii* infection and gene expression in brain cells [35,78]. The effect of CXCR3 deficiency was probably compensated for by the strong response occurring via the TLR2/MyD88 pathway. This may explain why no survival difference was observed between our WT and KO mice. However, the body weight difference that we saw during the chronic infection phase suggests that the lack of CXCR3 in the KO mice impaired their recovery from the damage inflicted by the acute infection.

Taken together, our data support a role for CXCR3 in both astrocytes and microglia that involves immune response triggering against *T. gondii*. The findings from this study will help to further current understanding of the mechanism controlling *T. gondii* infection via the chemokine–chemokine receptor signaling pathway in the brain. Further understanding could lead to future development of treatment and prevention of brain toxoplasmosis, for example, by activation of immune pathways against *T. gondii* through CXCR3 and its related genes.

## Figures and Tables

**Table 1:** Top five GO terms overrepresented in DEGs whose upregulation during *T. gondii* infection was impaired by CXCR3-deficiency. DEGs, differentially expressed genes; GO, gene ontology; FDR, false discovery rate.

Cell Type	Accession NO.	# DEGs	# Reference Genes	GO Term	FDR
Astrocyte	GO:0019882	10	96	antigen processing and presentation	0
	GO:0002376	42	2306	immune system process	1.8 × 10^−17^
	GO:0006952	32	1432	defense response	7.0 × 10^−15^
	GO:0006955	31	1316	immune response	8.5 × 10^−15^
	GO:0045087	24	714	innate immune response	4.1 × 10^−14^
Microglia	GO:0006954	18	647	inflammatory response	3.6 × 10^−7^
	GO:0001819	14	402	positive regulation of cytokine production	3.5 × 10^−6^
	GO:0006955	22	1316	immune response	1.0 × 10^−5^
	GO:0001817	16	635	regulation of cytokine production	1.0 × 10^−5^
	GO:0001816	16	707	cytokine production	4.1 × 10^−5^

## Data Availability

The data presented in this study are available on request from the corresponding author.

## Supplementary Materials

The following are available online at https://www.mdpi.com/article/10.3390/microorganisms9112340/s1, Figure S1. Heatmap of clinical scores of the hair coat of *T. gondii*-infected mice. Figure S2: Representative images of the brain tissue of *T. gondii*-infected CXCR3KO mice. Figure S3: Number of cysts distributed in each part of the brain, Figure S4: The numbers of T cells in the cerebral cortex of *T. gondii*-infected mice. Figure S5: Gene expression in primary glial cells infected by *T. gondii*, Table S1: Genes up/downregulated just by CXCR3-deficiency regardless of *T. gondii* infection, Table S2: Primer sequences used in RT-qPCR, Table S3: Detailed expression data for DEGs whose upregulation during *T. gondii* infection was impaired by CXCR3-deficiency, Table S4: Detailed data for top 10 GO terms overrepresented in the DEGs whose upregulation during *T. gondii* infection was impaired by CXCR3-deficiency in astrocytes and microglia, Table S5: Top 10 KEGG pathways enriched in the DEGs whose upregulation during *T. gondii* infection was impaired by CXCR3-deficiency in astrocytes and microglia.

## References

[1] Jones J.L., Dubey J.P. (2012). Foodborne Toxoplasmosis. Clin. Infect. Dis..

[2] Montoya J., Liesenfeld O. (2004). Toxoplasmosis. Lancet.

[3] Jones J., Lopez A., Wilson M. (2003). Congenital Toxoplasmosis. Am. Fam. Physician.

[4] Basavaraju A. (2016). Toxoplasmosis in HIV Infection: An Overview. Trop. Parasitol..

[5] Joiner K.A., Dubremetz J.F. (1993). *Toxoplasma Gondii*: A Protozoan for the Nineties. Infect. Immun..

[6] Blackwell T.S., Christman J.W. (1997). The Role of Nuclear Factor-Kappa B in Cytokine Gene Regulation. Am. J. Respir. Cell Mol. Biol..

[7] Shapira S., Speirs K., Gerstein A., Caamano J., Hunter C.A. (2002). Suppression of NF-ΚB Activation by Infection with *Toxoplasma Gondii*. J. Infect. Dis..

[8] Rosowski E.E., Lu D., Julien L., Rodda L., Gaiser R.A., Jensen K.D.C., Saeij J.P.J. (2011). Strain-Specific Activation of the NF-ΚB Pathway by GRA15, a Novel *Toxoplasma Gondii* Dense Granule Protein. J. Exp. Med..

[9] Aliberti J. (2005). Host Persistence: Exploitation of Anti-Inflammatory Pathways by *Toxoplasma Gondii*. Nat. Rev. Immunol..

[10] Yarovinsky F. (2014). Innate Immunity to *Toxoplasma Gondii* Infection. Nat. Rev. Immunol..

[11] Lüder C.G.K., Giraldo-Velásquez M., Sendtner M., Gross U. (1999). *Toxoplasma gondii* in Primary Rat CNS Cells: Differential Contribution of Neurons, Astrocytes, and Microglial Cells for the Intracerebral Development and Stage Differentiation. Exp. Parasitol..

[12] Cabral C.M., Tuladhar S., Dietrich H.K., Nguyen E., MacDonald W.R., Trivedi T., Devineni A., Koshy A.A. (2016). Neurons Are the Primary Target Cell for the Brain-Tropic Intracellular Parasite *Toxoplasma Gondii*. PLoS Pathog..

[13] Hatten M.E., Liem R.K.H., Shelanski M.L., Mason C.A. (1991). Astroglia in CNS Injury. Glia.

[14] Araque A., Carmignoto G., Haydon P.G. (2001). Dynamic Signaling Between Astrocytes and Neurons. Annu. Rev. Physiol..

[15] Contreras-Ochoa C.O., Lagunas-Martínez A., Belkind-Gerson J., Correa D. (2012). Toxoplasma Gondii Invasion and Replication in Astrocyte Primary Cultures and Astrocytoma Cell Lines: Systematic Review of the Literature. Parasitol. Res..

[16] Hidano S., Randall L.M., Dawson L., Dietrich H.K., Konradt C., Klover P.J., John B., Harris T.H., Fang Q., Turek B. (2016). STAT1 Signaling in Astrocytes Is Essential for Control of Infection in the Central Nervous System. mBio.

[17] Kettenmann H., Hanisch U.-K., Noda M., Verkhratsky A. (2011). Physiology of Microglia. Physiol. Rev..

[18] Kreutzberg G.W. (1996). Microglia: A Sensor for Pathological Events in the CNS. Trends Neurosci..

[19] Dellacasa-Lindberg I., Fuks J.M., Arrighi R.B.G., Lambert H., Wallin R.P.A., Chambers B.J., Barragan A. (2011). Migratory Activation of Primary Cortical Microglia upon Infection with *Toxoplasma Gondii*. Infect. Immun..

[20] Tanaka S., Nishimura M., Ihara F., Yamagishi J., Suzuki Y., Nishikawa Y. (2013). Transcriptome Analysis of Mouse Brain Infected with *Toxoplasma Gondii*. Infect. Immun..

[21] Cole K.E., Strick C.A., Paradis T.J., Ogborne K.T., Loetscher M., Gladue R.P., Lin W., Boyd J.G., Moser B., Wood D.E. (1998). Interferon-Inducible T Cell Alpha Chemoattractant (I-TAC): A Novel Non-ELR CXC Chemokine with Potent Activity on Activated T Cells through Selective High Affinity Binding to CXCR3. J. Exp. Med..

[22] Heise C.E., Pahuja A., Hudson S.C., Mistry M.S., Putnam A.L., Gross M.M., Gottlieb P.A., Wade W.S., Kiankarimi M., Schwarz D. (2005). Pharmacological Characterization of CXC Chemokine Receptor 3 Ligands and a Small Molecule Antagonist. J. Pharmacol. Exp. Ther..

[23] Loetscher M., Loetscher P., Brass N., Meese E., Moser B. (1998). Lymphocyte-Specific Chemokine Receptor CXCR3: Regulation, Chemokine Binding and Gene Localization. Eur. J. Immunol..

[24] Klein R.S., Lin E., Zhang B., Luster A.D., Tollett J., Samuel M.A., Engle M., Diamond M.S. (2005). Neuronal CXCL10 Directs CD8+ T-Cell Recruitment and Control of West Nile Virus Encephalitis. J. Virol..

[25] Campanella G.S.V., Tager A.M., El Khoury J.K., Thomas S.Y., Abrazinski T.A., Manice L.A., Colvin R.A., Luster A.D. (2008). Chemokine Receptor CXCR3 and Its Ligands CXCL9 and CXCL10 Are Required for the Development of Murine Cerebral Malaria. Proc. Natl. Acad. Sci. USA.

[26] Cohen S.B., Maurer K.J., Egan C.E., Oghumu S., Satoskar A.R., Denkers E.Y. (2013). CXCR3-Dependent CD4^+^ T Cells Are Required to Activate Inflammatory Monocytes for Defense against Intestinal Infection. PLoS Pathog..

[27] Khan I.A., MacLean J.A., Lee F.S., Casciotti L., DeHaan E., Schwartzman J.D., Luster A.D. (2000). IP-10 Is Critical for Effector T Cell Trafficking and Host Survival in *Toxoplasma Gondii* Infection. Immunity.

[28] Norose K., Kikumura A., Luster A.D., Hunter C.A., Harris T.H. (2011). CXCL10 Is Required to Maintain T-Cell Populations and to Control Parasite Replication during Chronic Ocular Toxoplasmosis. Invest. Ophthalmol. Vis. Sci..

[29] Harris T.H., Banigan E.J., Christian D.A., Konradt C., Wojno E.D.T., Norose K., Wilson E.H., John B., Weninger W., Luster A.D. (2012). Generalized Lévy Walks and the Role of Chemokines in Migration of Effector CD8+ T Cells. Nature.

[30] Biber K., Dijkstra I., Trebst C., De Groot C.J.A., Ransohoff R.M., Boddeke H.W.G.M. (2002). Functional Expression of CXCR3 in Cultured Mouse and Human Astrocytes and Microglia. Neuroscience.

[31] Xia M.Q., Bacskai B.J., Knowles R.B., Qin S.X., Hyman B.T. (2000). Expression of the Chemokine Receptor CXCR3 on Neurons and the Elevated Expression of Its Ligand IP-10 in Reactive Astrocytes: In Vitro ERK1/2 Activation and Role in Alzheimer’s Disease. J. Neuroimmunol..

[32] Ambrosini E., Aloisi F. (2004). Chemokines and Glial Cells: A Complex Network in the Central Nervous System. Neurochem. Res..

[33] Terkawi M.A., Kameyama K., Rasul N.H., Xuan X., Nishikawa Y. (2013). Development of an Immunochromatographic Assay Based on Dense Granule Protein 7 for Serological Detection of *Toxoplasma Gondii* Infection. Clin. Vaccine Immunol..

[34] Schneider C.A., Rasband W.S., Eliceiri K.W. (2012). NIH Image to ImageJ: 25 Years of Image Analysis. Nat. Methods.

[35] Umeda K., Tanaka S., Ihara F., Yamagishi J., Suzuki Y., Nishikawa Y. (2017). Transcriptional Profiling of Toll-like Receptor 2-Deficient Primary Murine Brain Cells during *Toxoplasma Gondii* Infection. PLoS ONE.

[36] Fischer H.G., Nitzgen B., Germann T., Degitz K., Däubener W., Hadding U. (1993). Differentiation Driven by Granulocyte-Macrophage Colony-Stimulating Factor Endows Microglia with Interferon-Gamma-Independent Antigen Presentation Function. J. Neuroimmunol..

[37] Kobayashi K., Umeda K., Ihara F., Tanaka S., Yamagishi J., Suzuki Y., Nishikawa Y. (2019). Transcriptome Analysis of the Effect of C-C Chemokine Receptor 5 Deficiency on Cell Response to *Toxoplasma Gondii* in Brain Cells. BMC Genom..

[38] Love M.I., Huber W., Anders S. (2014). Moderated Estimation of Fold Change and Dispersion for RNA-Seq Data with DESeq2. Genome Biol..

[39] McCarthy D.J., Chen Y., Smyth G.K. (2012). Differential Expression Analysis of Multifactor RNA-Seq Experiments with Respect to Biological Variation. Nucleic Acids Res..

[40] Robinson M.D., McCarthy D.J., Smyth G.K. (2010). EdgeR: A Bioconductor Package for Differential Expression Analysis of Digital Gene Expression Data. Bioinformatics.

[41] Tang M., Sun J., Shimizu K., Kadota K. (2015). Evaluation of Methods for Differential Expression Analysis on Multi-Group RNA-Seq Count Data. BMC Bioinform..

[42] Young M.D., Wakefield M.J., Smyth G.K., Oshlack A. (2010). Gene Ontology Analysis for RNA-Seq: Accounting for Selection Bias. Genome Biol..

[43] Carlson M. (2019). Org. Mm. Eg. Db: Genome Wide Annotation for Mouse. https://bioconductor.org/packages/release/data/annotation/html/org.Mm.eg.db.html.

[44] Kanehisa M., Sato Y., Kawashima M., Furumichi M., Tanabe M. (2016). KEGG as a Reference Resource for Gene and Protein Annotation. Nucleic Acids Res..

[45] Yu G., Wang L.-G., Han Y., He Q.-Y. (2012). ClusterProfiler: An R Package for Comparing Biological Themes Among Gene Clusters. OMICS A J. Integr. Biol..

[46] Bonfá G., Benevides L., Souza M.d.C., Fonseca D.M., Mineo T.W.P., Rossi M.A., Silva N.M., Silva J.S., de Barros Cardoso C.R. (2014). CCR5 Controls Immune and Metabolic Functions during *Toxoplasma Gondii* Infection. PLoS ONE.

[47] Xie F., Xiao P., Chen D., Xu L., Zhang B. (2012). MiRDeepFinder: A MiRNA Analysis Tool for Deep Sequencing of Plant Small RNAs. Plant Mol. Biol..

[48] Aviles H., Stiles J., O’Donnell P., Orshal J., Leid J., Sonnenfeld G., Monroy F. (2008). Kinetics of Systemic Cytokine and Brain Chemokine Gene Expression in Murine *Toxoplasma* Infection. J. Parasitol..

[49] Ferreira C.P., Cariste L.d.M., Noronha I.H., Durso D.F., Lannes-Vieira J., Bortoluci K.R., Ribeiro D.A., Golenbock D., Gazzinelli R.T., Vasconcelos J.R.C. (2020). de CXCR3 Chemokine Receptor Contributes to Specific CD8+ T Cell Activation by PDC during Infection with Intracellular Pathogens. PLoS Negl. Trop. Dis..

[50] Vasquez R.E., Soong L. (2006). CXCL10/Gamma Interferon-Inducible Protein 10-Mediated Protection against *Leishmania Amazonensis* Infection in Mice. Infect. Immun..

[51] Vasquez R.E., Xin L., Soong L. (2008). Effects of CXCL10 on Dendritic Cell and CD4+ T-Cell Functions during *Leishmania Amazonensis* Infection. Infect. Immun..

[52] Rappert A., Bechmann I., Pivneva T., Mahlo J., Biber K., Nolte C., Kovac A.D., Gerard C., Boddeke H.W.G.M., Nitsch R. (2004). CXCR3-Dependent Microglial Recruitment Is Essential for Dendrite Loss after Brain Lesion. J. Neurosci..

[53] Aksoy M.O., Yang Y., Ji R., Reddy P.J., Shahabuddin S., Litvin J., Rogers T.J., Kelsen S.G. (2006). CXCR3 Surface Expression in Human Airway Epithelial Cells: Cell Cycle Dependence and Effect on Cell Proliferation. Am. J. Physiol. Lung Cell Mol. Physiol..

[54] Silva N.M., Vieira J.C.M., Carneiro C.M., Tafuri W.L. (2009). *Toxoplasma gondii*: The Role of IFN-Gamma, TNFRp55 and INOS in Inflammatory Changes during Infection. Exp. Parasitol..

[55] Cabral G.R.d.A., Wang Z.T., Sibley L.D., DaMatta R.A. (2018). Inhibition of Nitric Oxide Production in Activated Macrophages Caused by *Toxoplasma Gondii* Infection Occurs by Distinct Mechanisms in Different Mouse Macrophage Cell Lines. Front. Microbiol..

[56] Butcher B.A., Fox B.A., Rommereim L.M., Kim S.G., Maurer K.J., Yarovinsky F., Herbert D.R., Bzik D.J., Denkers E.Y. (2011). *Toxoplasma gondii* Rhoptry Kinase ROP16 Activates STAT3 and STAT6 Resulting in Cytokine Inhibition and Arginase-1-Dependent Growth Control. PLoS Pathog..

[57] Yamamoto M., Okuyama M., Ma J.S., Kimura T., Kamiyama N., Saiga H., Ohshima J., Sasai M., Kayama H., Okamoto T. (2012). A Cluster of Interferon-γ-Inducible P65 GTPases Plays a Critical Role in Host Defense against *Toxoplasma Gondii*. Immunity.

[58] Shenoy A.R., Wellington D.A., Kumar P., Kassa H., Booth C.J., Cresswell P., MacMicking J.D. (2012). GBP5 Promotes NLRP3 Inflammasome Assembly and Immunity in Mammals. Science.

[59] Fujiwara Y., Hizukuri Y., Yamashiro K., Makita N., Ohnishi K., Takeya M., Komohara Y., Hayashi Y. (2016). Guanylate-Binding Protein 5 Is a Marker of Interferon-γ-Induced Classically Activated Macrophages. Clin. Transl. Immunol..

[60] Matta S.K., Patten K., Wang Q., Kim B.-H., MacMicking J.D., Sibley L.D. (2018). NADPH Oxidase and Guanylate Binding Protein 5 Restrict Survival of Avirulent Type III Strains of *Toxoplasma Gondii* in Naive Macrophages. mBio.

[61] Takeuchi O., Hoshino K., Akira S. (2000). Cutting Edge: TLR2-Deficient and MyD88-Deficient Mice Are Highly Susceptible to *Staphylococcus Aureus* Infection. J. Immunol..

[62] Thoma-Uszynski S., Stenger S., Takeuchi O., Ochoa M.T., Engele M., Sieling P.A., Barnes P.F., Rollinghoff M., Bolcskei P.L., Wagner M. (2001). Induction of Direct Antimicrobial Activity through Mammalian Toll-like Receptors. Science.

[63] Sasaki I., Hoshino K., Sugiyama T., Yamazaki C., Yano T., Iizuka A., Hemmi H., Tanaka T., Saito M., Sugiyama M. (2012). Spi-B Is Critical for Plasmacytoid Dendritic Cell Function and Development. Blood.

[64] Silver J.S., Stumhofer J.S., Passos S., Ernst M., Hunter C.A. (2011). IL-6 Mediates the Susceptibility of Gp130 Hypermorphs to *Toxoplasma Gondii*. J. Immunol..

[65] Jebbari H., Roberts C.W., Ferguson D.J., Bluethmann H., Alexander J. (1998). A Protective Role for IL-6 during Early Infection with *Toxoplasma Gondii*. Parasite Immunol..

[66] Yap G., Pesin M., Sher A. (2000). Cutting Edge: IL-12 Is Required for the Maintenance of IFN-Gamma Production in T Cells Mediating Chronic Resistance to the Intracellular Pathogen, *Toxoplasma Gondii*. J. Immunol..

[67] Sher A., Collazzo C., Scanga C., Jankovic D., Yap G., Aliberti J. (2003). Induction and Regulation of IL-12-Dependent Host Resistance to *Toxoplasma Gondii*. Immunol. Res..

[68] Peng B.-W., Lin J., Zhang T. (2008). *Toxoplasma gondii* Induces Prostaglandin E2 Synthesis in Macrophages via Signal Pathways for Calcium-Dependent Arachidonic Acid Production and PKC-Dependent Induction of Cyclooxygenase-2. Parasitol. Res..

[69] Pereira A.C.A., Silva R.J., Franco P.S., de Oliveira Gomes A., Souza G., Milian I.C.B., Ribeiro M., Rosini A.M., Guirelli P.M., Ramos E.L.P. (2019). Cyclooxygenase (COX)-2 Inhibitors Reduce *Toxoplasma Gondii* Infection and Upregulate the Pro-Inflammatory Immune Response in Calomys Callosus Rodents and Human Monocyte Cell Line. Front. Microbiol..

[70] Ha M., Jeong H., Roh J.S., Lee B., Lee D., Han M.-E., Oh S.-O., Sohn D.H., Kim Y.H. (2019). VNN3 Is a Potential Novel Biomarker for Predicting Prognosis in Clear Cell Renal Cell Carcinoma. Anim. Cells Syst..

[71] Hamilton T.A., Bredon N., Ohmori Y., Tannenbaum C.S. (1989). IFN-Gamma and IFN-Beta Independently Stimulate the Expression of Lipopolysaccharide-Inducible Genes in Murine Peritoneal Macrophages. J. Immunol..

[72] Der S.D., Zhou A., Williams B.R.G., Silverman R.H. (1998). Identification of Genes Differentially Regulated by Interferon α, β, or γ Using Oligonucleotide Arrays. PNAS.

[73] Schneider W.M., Chevillotte M.D., Rice C.M. (2014). Interferon-Stimulated Genes: A Complex Web of Host Defenses. Annu. Rev. Immunol..

[74] Smit M.J., Verdijk P., van der Raaij-Helmer E.M.H., Navis M., Hensbergen P.J., Leurs R., Tensen C.P. (2003). CXCR3-Mediated Chemotaxis of Human T Cells Is Regulated by a Gi- and Phospholipase C-Dependent Pathway and Not via Activation of MEK/P44/P42 MAPK nor Akt/PI-3 Kinase. Blood.

[75] Shahabuddin S., Ji R., Wang P., Brailoiu E., Dun N., Yang Y., Aksoy M.O., Kelsen S.G. (2006). CXCR3 Chemokine Receptor-Induced Chemotaxis in Human Airway Epithelial Cells: Role of P38 MAPK and PI3K Signaling Pathways. Am. J. Physiol. - Cell Physiol..

[76] Willox I., Mirkina I., Westwick J., Ward S.G. (2010). Evidence for PI3K-Dependent CXCR3 Agonist-Induced Degranulation of Human Cord Blood-Derived Mast Cells. Mol. Immunol..

[77] Yin M., Shen Z., Yang L., Zheng W., Song H. (2019). Protective Effects of CXCR3/HO-1 Gene-modified BMMSCs on Damaged Intestinal Epithelial Cells: Role of the P38-MAPK Signaling Pathway. Int. J. Mol. Med..

[78] Mun H.-S., Aosai F., Norose K., Chen M., Piao L.-X., Takeuchi O., Akira S., Ishikura H., Yano A. (2003). TLR2 as an Essential Molecule for Protective Immunity against *Toxoplasma Gondii* Infection. Int. Immunol..

