# Supplementary material for: Transcriptomic Analysis of the Effects of Chemokine Receptor CXCR3 Deficiency on Immune Responses in the Mouse Brain during Toxoplasma gondii Infection
Source: Microorganisms. 2021 Nov 12;9(11):2340. doi: 10.3390/microorganisms9112340 (PMC8620038; doi:10.3390/microorganisms9112340)
Supplement: Supplementary file 1 [file microorganisms-09-02340-s001.zip › CXCR3_Supplementary_211031.pdf]

Supplementary Material

1 Supplementary Figures and Tables

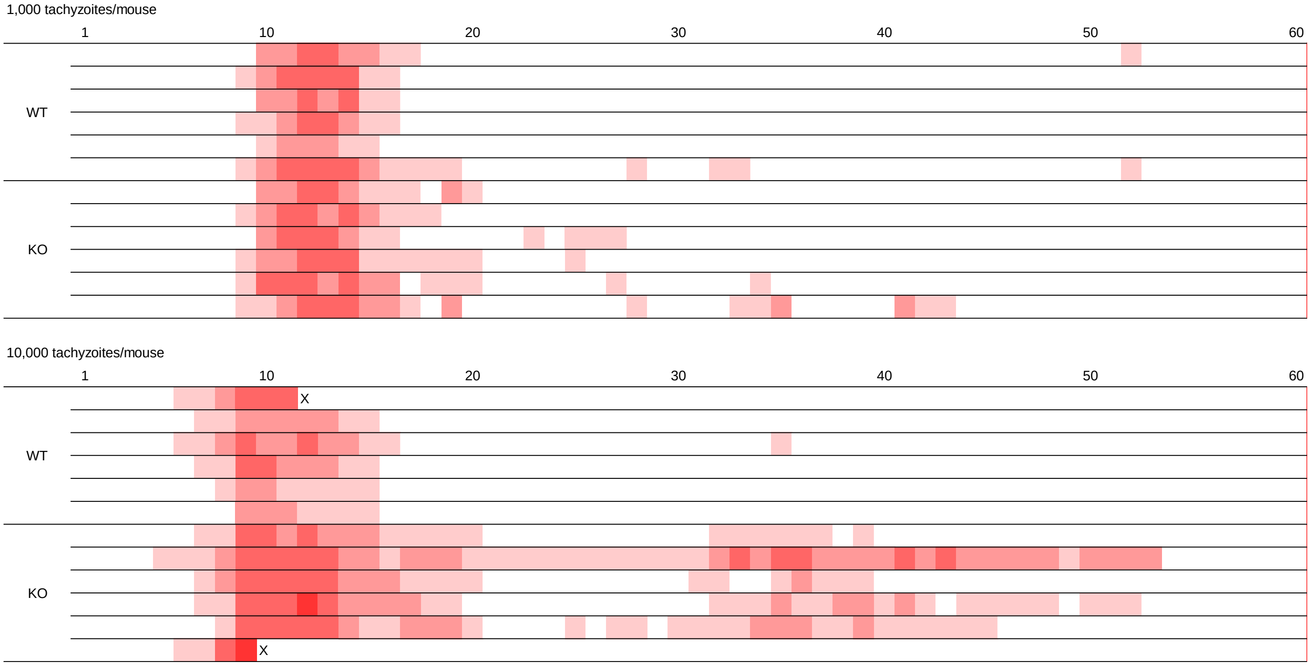

Figure S1. Heatmap of clinical scores of the hair coat of *T. gondii*-infected mice. WT and CXCR3KO mice were injected with 1,000 or 10,000 tachyzoites. Their hair coat was monitored daily for 60 days, and the poorness was scored on a scale of 0 to 4. X represents the day when the mouse died.

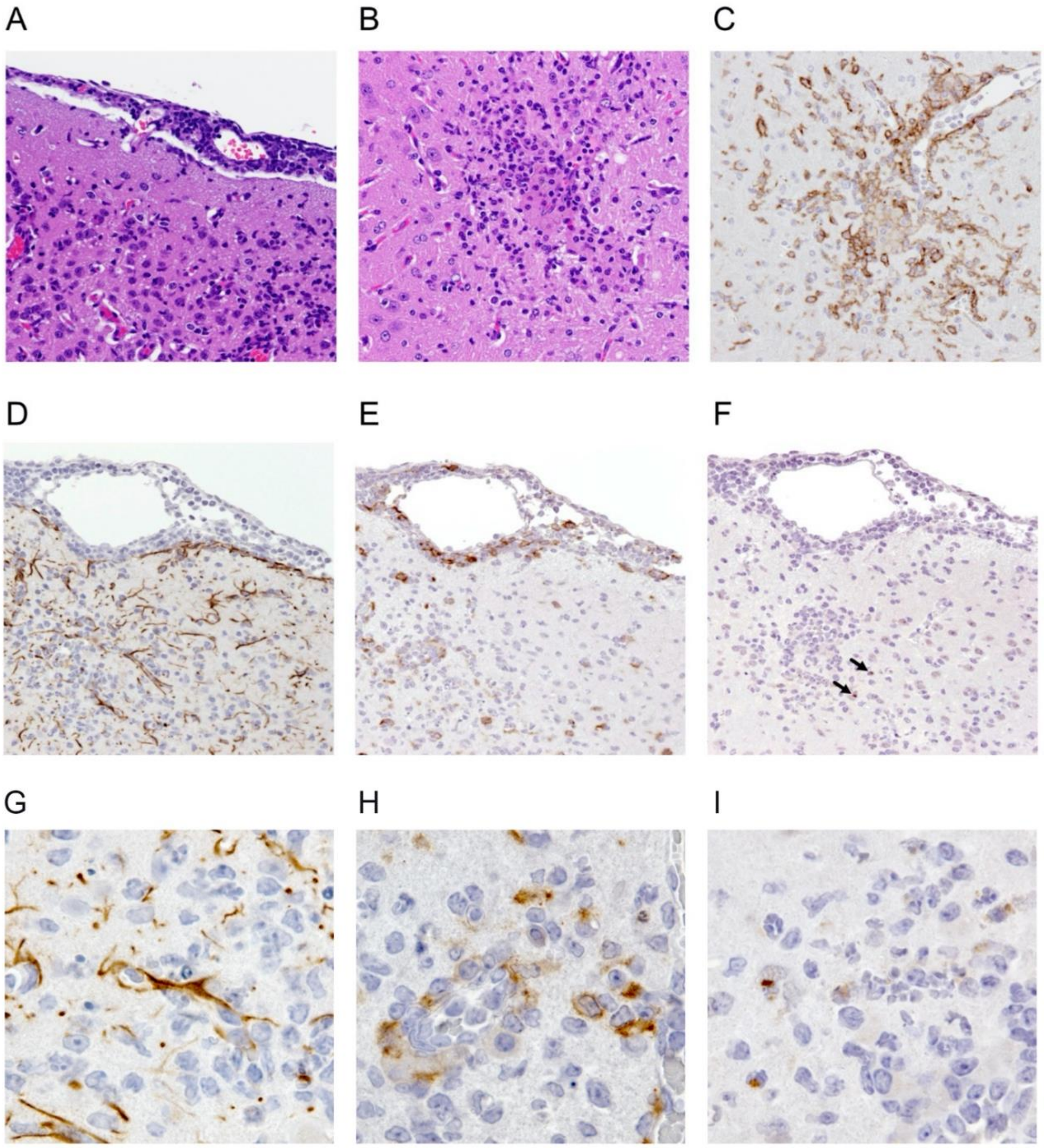

Figure S2. Representative images of the brain tissue of *T. gondii*-infected CXCR3KO mice. (A) Nonsuppurative meningoencephalitis. HE stained. (B, C) Perivascular cuffing and glial cell proliferation. B, HE stained; C, anti-IBA1. (D, E, F, G, H, I) Grial cells around *T. gondii*-infected area. G, H, and I are higher magnification of D, E, and F. D, G anti-GFAP; E, H anti-IBA1; F, I anti-*T. gondii*. Arrow: Tachyzoite of *T. gondii*. All of these images are from CXCR3KO mice on day 60 post infection of 10,000 tachyzoites per mouse.

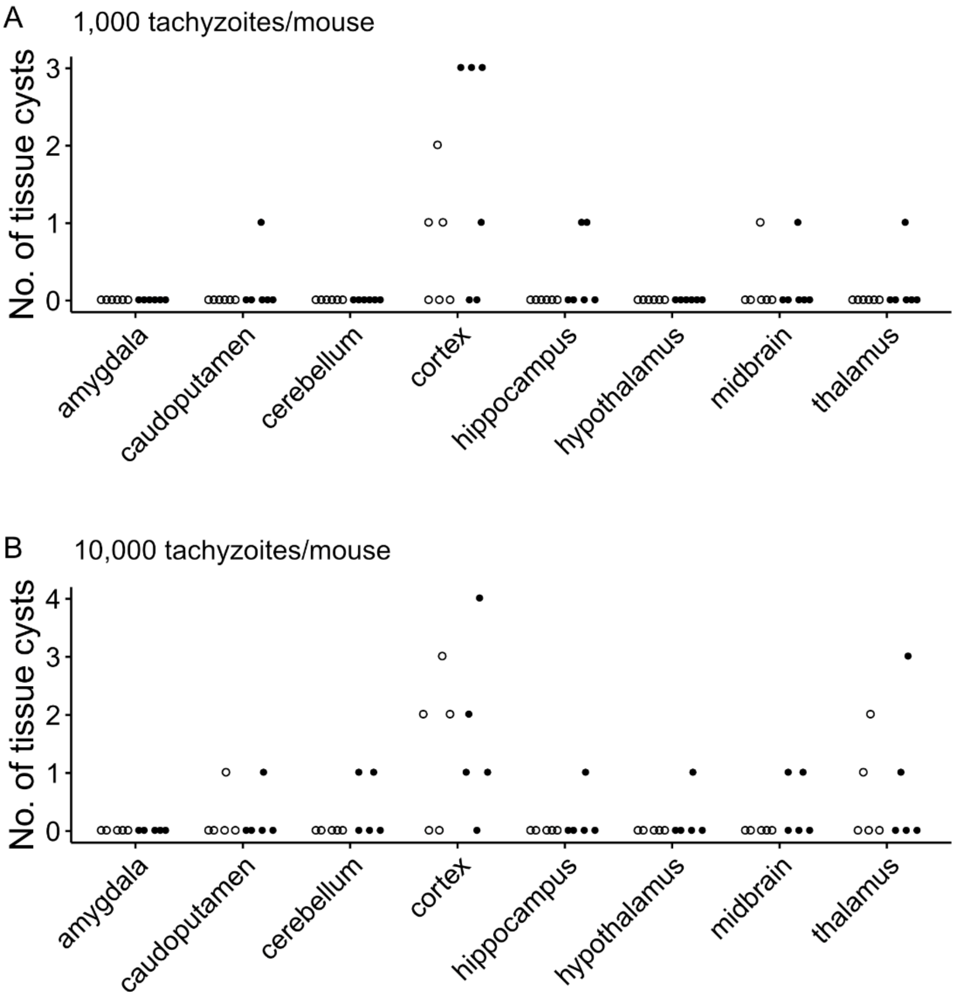

Figure S3. Number of cysts distributed in each part of the brain. WT (open circles) and CXCR3KO (closed circles) mice were subjected to histopathological processing on day 60 post infection. After the immunohistochemistry analysis using an anti-*T. gondii* antibody, the cysts were counted in 10 images (×100 magnification) of the cortex, hippocampus, caudoputamen, amygdala, thalamus, hypothalamus, midbrain, and cerebellum. (A) 1,000 tachyzoites per mouse (n = 6 per group); (B) 10,000 tachyzoites per mouse (n = 5 per group).

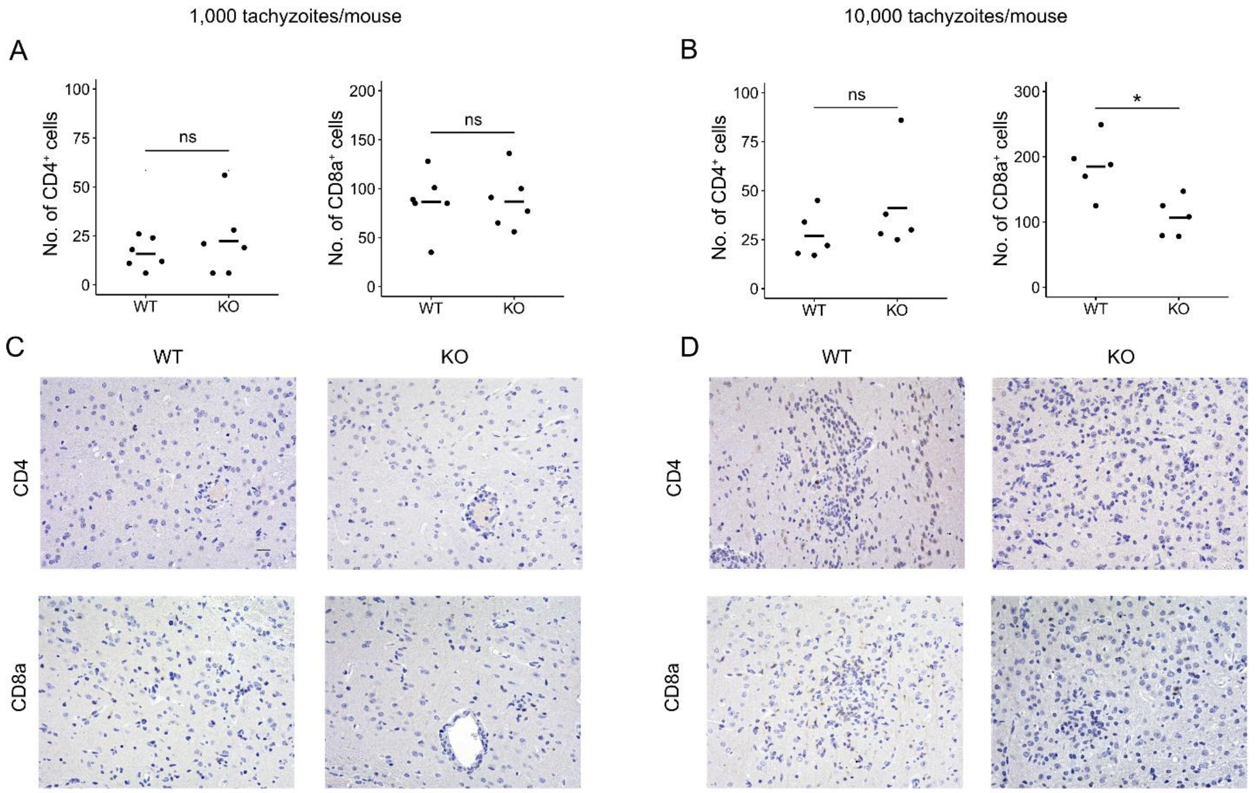

Figure S4: T cells in the cerebral cortex of *T. gondii*-infected mice. The brains from WT and CXCR3KO mice surviving on day 60 post infection were subjected to histopathological processing and immunohistochemistry against CD4 and CD8a. (A, B) Numbers of positive cells in 10 images (×100 magnification). ns, not significant; $*p < 0.05$, two-tailed unpaired Student's t-test. Horizontal bars represent the mean values. (C, D) Representative immunohistochemical images. (A, C) 1,000 tachyzoites per mouse (n = 6 per group); (B, D) 10,000 tachyzoites per mouse (n = 5 per group). While the numbers of $CD4^+$ T cell showed no significant differences between the genotypes, the numbers of $CD8a^+$ T cell in the brains of mice injected with 10,000 tachyzoites were significantly higher in the WT mice than in the CXCR3KO mice.

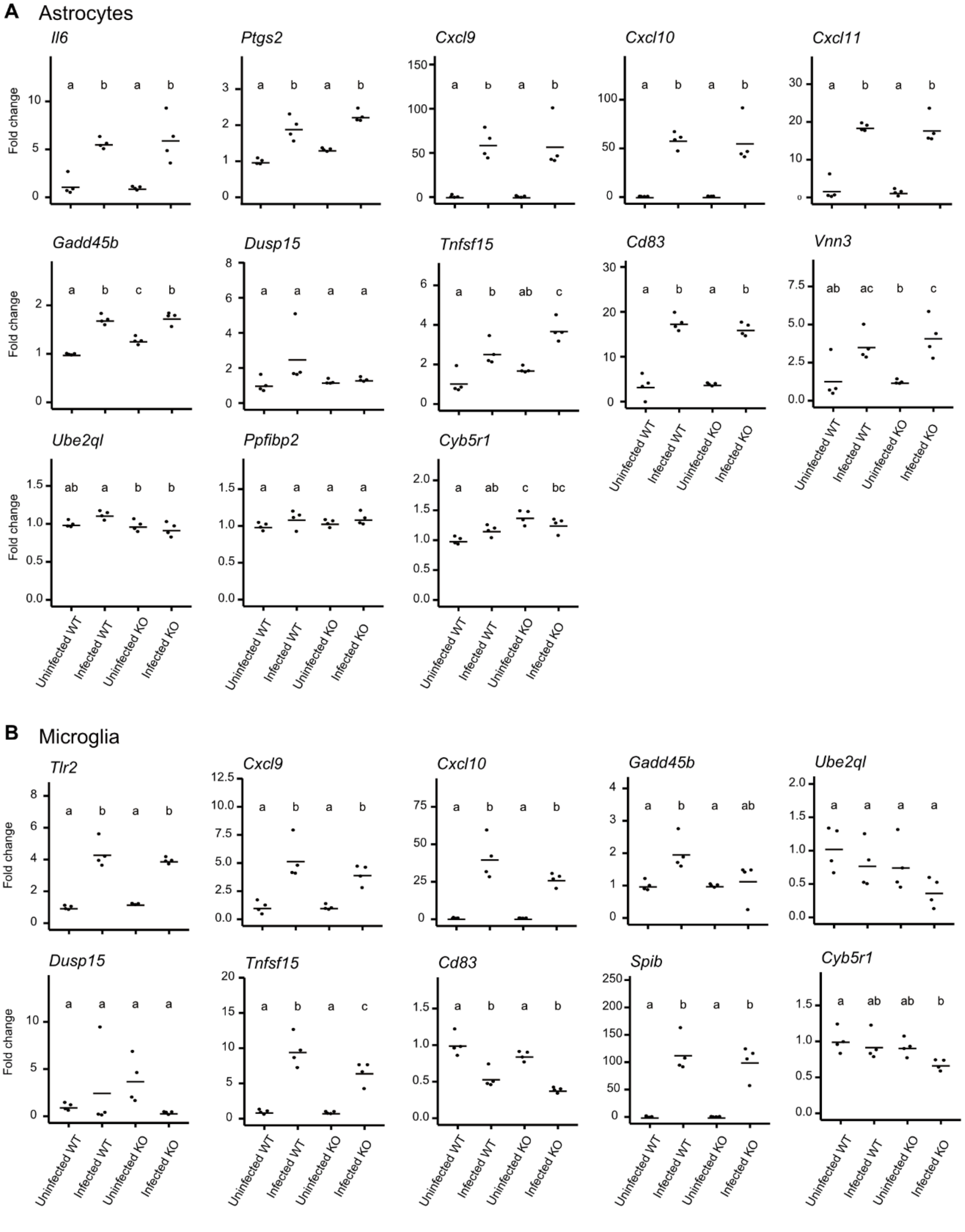

Figure S5. Gene expression in primary glial cells infected by *T. gondii*. Genes with no significant interactions between mouse genotype and infection in a two-way ANOVA are shown. (A) Astrocytes; (B) Microglia. Horizontal bars represent the mean value.

Table S1. Genes up/downregulated just by CXCR3-deficiency regardless of *T. gondii* infection. DEGs were identified by comparing between the uninfected WT and uninfected CXCR3KO mice, with a threshold of 2-fold change and <0.05 FDR.

Table S2. Primer sequences used in RT-qPCR.

Table S3. Detailed expression data for DEGs whose upregulation during *T. gondii* infection was impaired by CXCR3-deficiency.

Table S4. Detailed data for top 10 GO terms overrepresented in the DEGs whose upregulation during *T. gondii* infection was impaired by CXCR3-deficiency in astrocytes and microglia.

Table S5. Top10 KEGG pathways enriched in the DEGs whose upregulation during *T. gondii* infection was impaired by CXCR3-deficiency in astrocytes and microglia.
